# Comparison of cardiovascular magnetic resonance of late gadolinium enhancement and diastolic wall thickness to predict recovery of left ventricular function after coronary artery bypass surgery

**DOI:** 10.1186/1532-429X-10-41

**Published:** 2008-09-22

**Authors:** Rungroj Krittayaphong, Pansak Laksanabunsong, Adisak Maneesai, Pairash Saiviroonporn, Suthipol Udompunturak, Vithaya Chaithiraphan

**Affiliations:** 1 Division of Cardiology, Department of Medicine, Faculty of Medicine, Siriraj Hospital, Mahidol University, Bangkok, Thailand; 2 Department of Surgery, Faculty of Medicine, Siriraj Hospital, Mahidol University, Bangkok, Thailand; 3 Department of Radiology, Faculty of Medicine, Siriraj Hospital, Mahidol University, Bangkok, Thailand; 4 Department of Research Promotion, Faculty of Medicine, Siriraj Hospital, Mahidol University, Bangkok, Thailand; 5 Her Majesty Cardiac Center, Faculty of Medicine, Siriraj Hospital, Mahidol University, Bangkok, Thailand

## Abstract

**Background:**

The objective was to compare the value of late gadolinium enhancement (LGE) and end-diastolic wall thickness (EDWT) assessed by cardiovascular magnetic resonance (CMR) in predicting recovery of left ventricular function after coronary artery bypass surgery (CABG).

**Methods:**

We enrolled patients with coronary artery disease and left ventricular ejection fraction < 45% who were scheduled for CABG. Regional contractility was assessed by cine CMR at baseline and 4 months after CABG. EDWT and LGE were assessed at baseline. Predictors for improvement of regional contractility were analyzed.

**Results:**

We studied 46 men and 4 women with an average age of 61 years. Baseline left ventricular ejection fraction was 37 ± 13%. A total of 2,020 myocardial segments were analyzed. Abnormal wall motion and the LGE area were detected in 1,446 segments (71.6%) and 1,196 segments (59.2%) respectively. Wall motion improvement was demonstrated in 481 of 1,227 segments (39.2%) that initially had wall motion abnormalities at baseline. Logistic regression analysis showed that the LGE area, EDWT and resting wall motion grade predicted wall motion improvement. Comparison of Receiver-Operator-Characteristic (ROC) curves demonstrated that the LGE area was the most important predictor (p < 0.001). Adding information from LGE to the EDWT can decrease the number of false predictions by EDWT alone from 483 to 127 segments.

**Conclusion:**

LGE and EDWT are independent predictors for functional recovery after revascularization. However, LGE appears to be a more important factor and independent of EDWT.

## Introduction

Myocardial hibernation is the state of left ventricular systolic dysfunction from chronic myocardial ischemia. Revascularization usually results in an improvement of this condition [1,2]. However, it may be irreversible if the myocardium is permanently damaged. Accurate selection of revascularization candidates is important since coronary artery bypass surgery (CABG) has higher morbidity and mortality in patients with more severe left ventricular dysfunction, especially in those without significant myocardial viability [3]. On the other hand, CABG can be life-saving, as the annual mortality rate is more than 4-fold greater in patients with a significant viable myocardium who were treated medically compared to those who underwent revascularization [3].

End-diastolic wall thickness (EDWT) is an important parameter of myocardial viability that can predict recovery of myocardial function [3,4]. In clinical practice thinning of the myocardial wall as observed from echocardiograms in patients with coronary disease usually raises concerns about the possibility of recovery in regional function after CABG. Cardiovascular magnetic resonance (CMR) has been shown to be an accurate technique for the assessment of global and regional ventricular dysfunction and myocardial viability by late gadolinium enhancement (LGE) and EDWT assessment [5]. It has been shown that the likelihood of improvement in regional contractility after CABG decreased as the extent of the LGE area increased [6]. Although LGE and assessment of EDWT [4,7] can be used to predict recovery of wall motion after CABG, there has been no data comparing these 2 parameters in the prediction of wall motion improvement after CABG.

The primary objective of this study was to assess the accuracy of CMR in determining the recovery of abnormal wall motion after CABG by measuring the extent of the LGE area and EDWT. A secondary objective was to assess predictors of global improvement in left ventricular ejection fraction (LVEF).

## Methods

### Study population

We studied male and female patients 30–80 years of age who had coronary artery disease (CAD) confirmed by a coronary angiogram with left ventricular dysfunction defined as LVEF of < 45%, stable symptoms, and were scheduled for CABG. Patients were excluded if they had contraindications for CMR (such as those with a ferromagnetic prosthesis, pacemakers or an internal defibrillator implantation), previous CABG, an allergy to gadolinium, were pregnant, had unstable hemodynamics, or had a requirement for urgent revascularization as well as those with a history of acute myocardial infarction within 3 months.

### Study procedures

This study was approved by the Ethics Committee of Siriraj Hospital. Informed consent was obtained prior to participation in all patients. Baseline demographic data and patient characteristics as well as ECG data were recorded. The presence of a Q-wave from the ECG was evaluated by the Minnesota code criteria [8]. CMR was performed for the assessment of overall left ventricular function, regional wall motion, EDWT and LGE at baseline. CMR was performed 4 months after CABG to assess overall cardiac function and wall motion improvement. Recording parameters also included CABG outcomes and complications, improvement of symptoms, hospitalization and clinical events after CABG also were recorded.

### CMR protocol

CMR was performed with the Gyroscan NT Intera 1.5 Tesla Philips scanner (Philips Medical Systems, Best, the Netherlands). After obtaining the scout images, and spin echo for structural evaluation, the functional study was performed with the 2D-balanced-fast-field echo (FFE) technique in the vertical long axis, 4-chamber view, and multiple slice short axis view covering the whole left ventricle. Cine images were obtained by using cardiac gated sequences. Parameters for functional images were as follows: repetition time/echo time/number of excitations (TR/TE/NEX) = 3.7/1.8/2, 390 × 312 mm field of view, 256 × 240 matrix, 1.52 × 1.3 reconstruction pixel, 8 mm slice thickness, and 70 degree flip angle.

LGE images were acquired 7–10 minutes after a 0.2 mmol/kg gadolinium injection (Magnevist, Schering AG, Berlin, Germany). The inversion time was adjusted to null normal myocardium. The viability study was assessed in the short axis view, 2 chamber and 4 chamber views. The acquisition of short axis views began at the level of mitral valve insertion and continued through the left ventricle. The LGE images were obtained with the same number of slices and same positions as the cine functional images on all views. The images were acquired with the use of 3D segmented-gradient-echo inversion-recovery sequence with TR/TE = 4.1/1.25 ms, 303 × 384 mm field of view, 240 × 256 matrix, 1.26 × 1.5 mm reconstruction pixel, 8 mm slice thickness, 15 degree flip angle, and 1.5 SENSitivity Encoding (SENSE) factor. The total study period took approximately 40 minutes.

### Analysis of CMR

Image analysis was performed on an independent workstation. Segmentation of each slice was performed according to the recommendation of the American Heart Association with the exclusion of segment 17 (most apical part)[9]. Segments with a suboptimal image quality were excluded from analysis. Wall motion of each myocardial segment before and after CABG was recorded by a 5-grade system as follows: 1 = normal, 2 = mild or moderate hypokinesia, 3 = severe hypokinesia, 4 = akinesia or 5 = dyskinesia.

The epicardial, endocardial contours and LGE area in each of the short-axis images were manually delineated. The presence and extent of a LGE area was divided into 5 grades: 0%, 1–25%, 26–50%, 51–75%, and 76–100% according to the extent of the area of LGE as a percentage to the myocardial area of each segment

All data were interpreted by 2 cardiologists blinded to the patient name and timing of CMR. Images before and after CABG in the same patient were separately analyzed. LGE images were read in a blinded fashion to cine CMR images. Any disagreement was solved by a consensus. LVEF was assessed by using end-systolic and end-diastolic volume calculated from the multiple slice short axis images.

Functional data assessment by CMR was repeated 4 months after CABG. Recovery of wall motion abnormality was defined as an improvement in wall motion abnormality by at least 1 grade.

Segmental analysis data from the first 20 patients were assessed for intraobserver and interobserver variability by the methods of Bland and Altman. Mean differences for intraobserver and interobserver variability of percentages of LGE area in each segment were 1.9% (limits of agreement ± 5.1%) and 2.8% (limits of agreement ± 9.2%). Assessment of Intraobserver and interobserver variability for EDWT revealed a mean difference of 0.07 mm (limits of agreement ± 0.7 mm) and 0.05 mm (limits of agreement ± 0.9 mm). Intra- and interobserver agreement for the presence of a LGE area were k = 0.94, p < 0.001 and k = 0.97, p < 0.001 respectively.

### Statistical analysis

Continuous variables were described as mean ± standard deviation (SD) and categorical variables were described as frequencies and percentages. A comparison of continuous variables was made by the unpaired t-test and comparison of categorical variables was made by the chi-square test. Chi-square test for trend and logistic regression analysis of 'enter' method with a repeated measure variable for the patient, to adjust for the non-independence of data, was used for the assessment of predicting the recovery of wall motion abnormality after CABG. Variables with a p value < 0.1 from the univariate analysis were selected for logistic regression analysis for both segmental wall motion improvement and global improvement in LVEF. Receiver-operating-characteristic (ROC) analysis was performed by the method of Metz [10] to assess the appropriate cut off value of the predictors derived from the logistic regression analysis and to acquire the area under the curve. The statistical significance of differences between the areas under ROC curves was evaluated by a univariate z-score test.

## Results

Fifty-two patients were screened. One patient was excluded due to recent myocardial infarction and another due to pacemaker implantation. Fifty patients were enrolled. There were 46 men and 4 women with an average age of 60.8 ± 9 years. Patients were enrolled during November 2003 to June 2005. Baseline characteristics are shown in Table 1. Thirty-three patients (66%) had a history of myocardial infarction. The average time after myocardial infarction was 36.3 ± 46.9 months.

**Table 1:** Baseline patient characteristics. Values are numbers (percentages) unless otherwise stated.

Characteristics	Number (%)
Male	46 (92)
Mean (SD) age (years)	60.8 (9)
Smoking	23 (46)
Hypercholesterolemia	46 (92)
Hypertension	30 (60)
History of myocardial infarction	33 (66)
History of percutaneous coronary intervention	8 (16)
History of heart failure	45 (90)
Presence of angina	43 (86)
Mean (SD) frequency of angina (per month)	31.8 (42)
NYHA classification	
I	1 (2)
II	23 (46)
III	26 (52)
Q-wave from ECG	22 (44)
Coronary angiogram	
2-vessel disease	9 (18)
At least 3-vessel disease	41 (82)
Mean (SD) left ventricular ejection fraction (%)	37.1 (12.8)

NYHA = New York Heart Association

### Results of baseline CMR

All patients had wall motion abnormalities from baseline CMR. Average LVEF was 37.1 ± 12.8%. An area of LGE was demonstrated in 49 patients (98%). A total of 2,020 myocardial segments were analyzed. Abnormal wall motion was detected in 1,446 segments (71.6%). An LGE area was demonstrated in 1,196 segments (59.2%). Grading of the LGE area and wall motion is shown in Table 2. Average EDWT was 5.9 ± 2.0 mm. Average EDWT was greater in segments without LGE compared to those with LGE (6.2 ± 1.9 versus 5.6 ± 2.0, p < 0.001).

**Table 2:** Characteristics of myocardial segments from baseline CMR

Characteristics	Number of segments (%)
Grading of LGE area	
0%	824 (40.8)
1–25%	201 (10)
26–50%	417 (20.6)
51–75%	340 (16.8)
76–100%	238 (11.8)
Grading of wall motion	
1 (normal)	574 (28.4)
2 (mild to moderate hypokinesia)	566 (28)
3 (severe hypokinesia)	476 (23.6)
4 (akinesia)	332 (16.4)
5 (dyskinesia or aneurysm)	72 (3.6)

### Outcomes of CABG

Fourteen patients (28.6%) had in-hospital complications which included atrial fibrillation in 4, acute renal failure in 2, bleeding that required re-operation in 3, compartment syndrome in 1, sepsis in 1, malignant ventricular arrhythmia in 1, delirium in 1, perioperative myocardial infarction in 1 and ischemic stroke in 1. Three patients (6.1%) died during hospital admission for CABG.

Three patients died within 4 months after CABG: 1 from ischemic stroke, 1 from a ruptured abdominal aortic aneurysm and 1 from ventricular tachyarrhythmia. Three patients required hospitalization during the 4 months after CABG for heart failure.

Compared to baseline, there was a significant decrease in frequency of angina (35.3 ± 43.6 versus 0.5 ± 1.8 per month, p < 0.001), use of sublingual nitroglycerin to relieve chest pain (27.7 ± 41.3 versus 0.1 ± 0.3 tablets per month, p < 0.001), and New York Heart Association class (2.52 ± 0.55 versus 1.48 ± 0.55, p < 0.001) after CABG.

### Prediction of wall motion improvement

**Figure 1:**
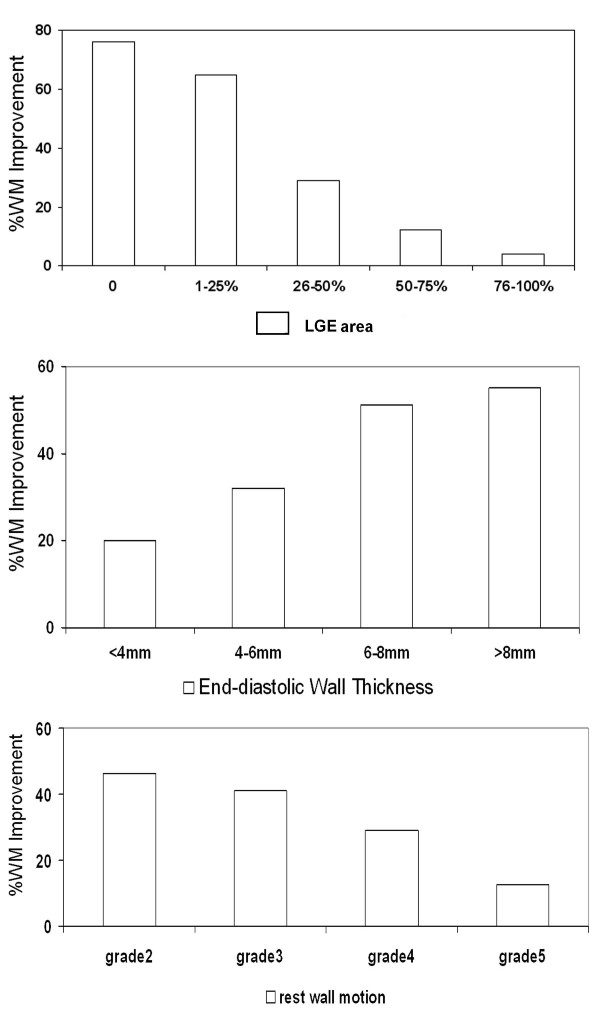
Relation of wall motion improvement and LGE area (upper), end-diastolic wall thickness (middle) and resting wall motion (lower)(WM = wall motion).

Follow-up CMR data was assessed in 44 patients. Comparisons of left ventricular size, volume and LVEF are shown in Table 3. There was a significant decrease in left ventricular end-diastolic diameter, end-systolic diameter, end-systolic volume and a significant increase in left ventricular stroke volume and LVEF. Considering only segments with baseline wall motion abnormality, the follow-up data showed wall motion improvement in 481 out of 1,227 segments (39.2%). Univariate analysis of parameters for the prediction of wall motion improvement after CABG is shown in Table 4. Figure 1 shows a bar graph of the relation of wall motion improvement with LGE area, EDWT and resting wall motion grade. The probability of wall motion improvement increased when the LGE area decreased (p < 0.001 for both) or the EDWT increased (p < 0.001) or the resting wall motion abnormality was less severe (p < 0.001).

**Table 3:** CMR parameters before and after CABG (n = 44)

	Baseline	After CABG	P value
LVDD (mm)	61.3 ± 8.3	59.2 ± 7.2	0.015
LVSD (mm)	51 ± 10.1	48.7 ± 11.8	0.035
LVEDV (ml)	182.8 ± 59.5	179.7 ± 70.8	0.469
LVESV (ml)	119.4 ± 60.1	110.7 ± 67.4	0.04
LVSV (ml)	63.3 ± 13.7	68.5 ± 16	0.006
LVEF (%)	37.9 ± 13.1	42.5 ± 15.3	0.001

LVDD = left ventricular diastolic diameter, LVDS = left ventricular systolic diameter, LVEDV = left ventricular end-diastolic volume, LVESV = left ventricular end-systolic volume, LVSV = left ventricular stroke volume, LVEF = left ventricular ejection fraction

**Table 4:** Univariate analysis of predictors for wall motion improvement after CABG (LGE – late gadolinium enhancement)

Parameters	Wall motion improvement		P value
		
	Yes N = 481	No N = 746	
LGE area (%)	15.2 ± 22	54.4 ± 31	< 0.001
LGE area/non-LGE area (%)	34.6 ± 105.3	142.8 ± 378.0	<0.001
EDWT (mm)	6.35 ± 1.78	5.41 ± 1.85	< 0.001
Resting wall motion grade	2.72 ± 0.79	3.04 ± 0.94	< 0.001

From the logistic regression analysis with the adjustment of the within patient factor, all of the 3 parameters were in the final equation as follows: LGE area (Odds ratio 0.92, 95% CI 0.91–0.93, p < 0.001), EDWT (Odds ratio 1.31, 95% CI 1.21–1.41, p < 0.001) and resting wall motion grade (Odds ratio 8.29, 95% CI 6.62–10.34, p < 0.001). This finding indicates that each factor is an independent predictor for segmental wall improvement. ROC curve of the LGE area in the prediction of wall motion improvement had the highest area under the curve of 0.837. By using the cut off level of 25%, sensitivity, specificity, positive and negative predictive values for the prediction of wall motion improvement were 74.2%, 83.2%, 62.6% and 81.1% respectively. The area under the curve of EDWT and resting wall motion grade were 0.647 and 0.591. At the cut off level of 5.5 mm, EDWT had sensitivity, specificity, positive and negative predictive values for the prediction of wall motion improvement were 70.1%, 54.6%, 49.8%, and 73.9% respectively. Resting wall motion grade, at the cut off level of grade 3, had sensitivity, specificity, positive and negative predictive values of 81.5%, 33.2%, 44.0% and 73.5% respectively.

**Figure 2:**
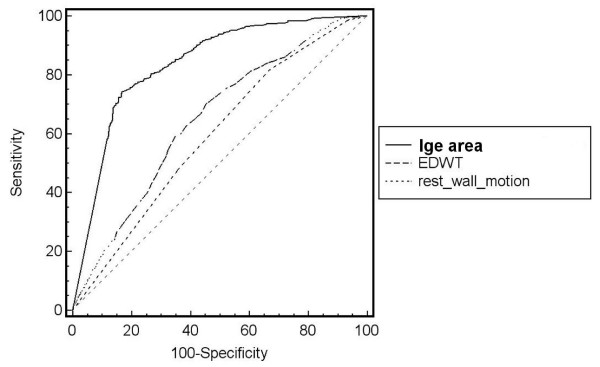
Comparison of ROC curves for the prediction of wall motion improvement by LGE area, end-diastolic wall thickness and resting wall motion.

**Figure 3:**
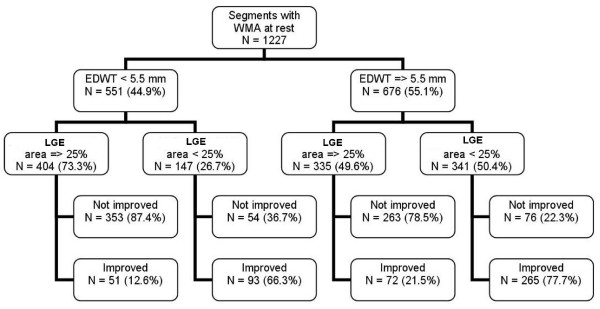
Diagram of the prediction of wall motion improvement by EDWT and LGE area (WMA = wall motion abnormality).

**Figure 4:**
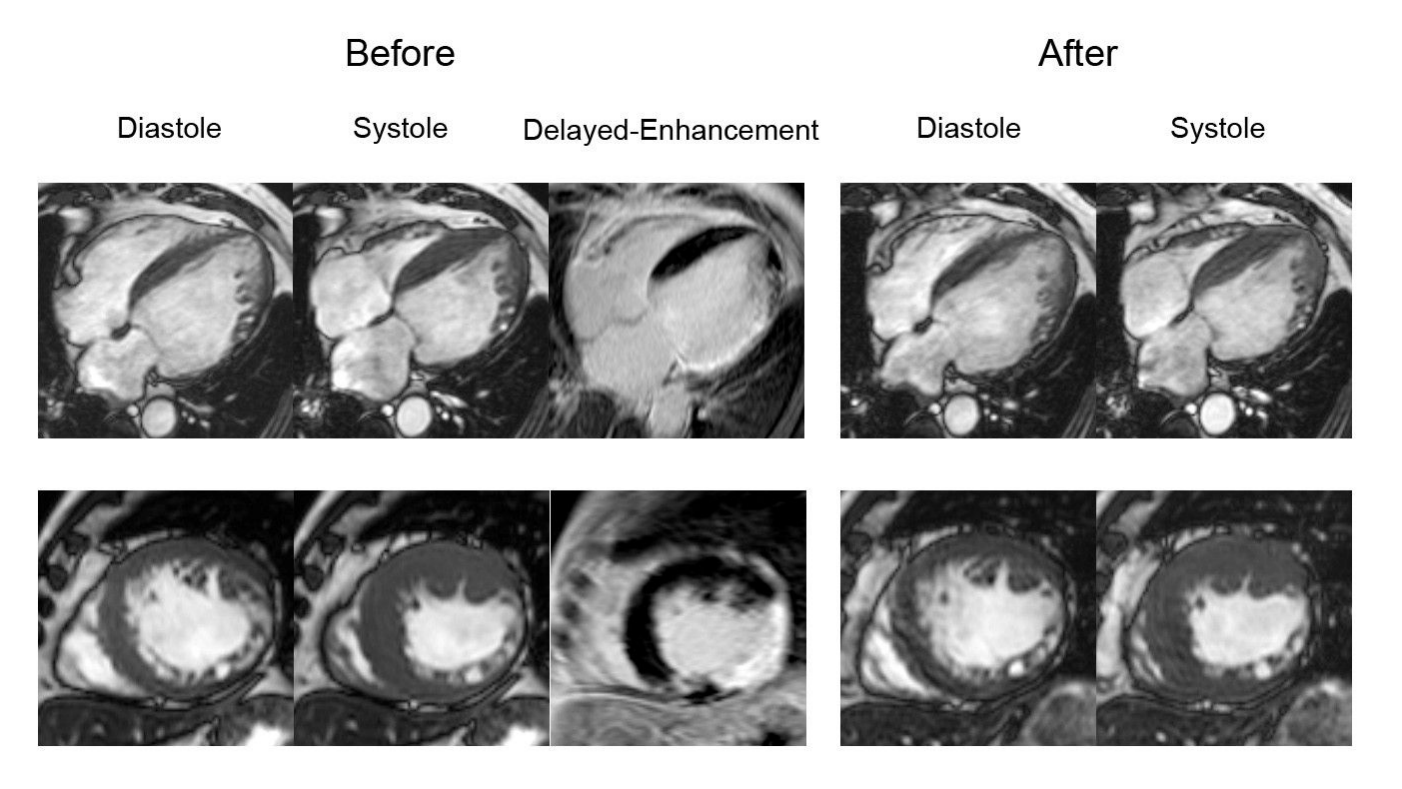
End-diastolic, end-systolic and LGE images before CABG and end-diastolic and end-systolic images after CABG of a patient with wall thinning and LGE area 76–100% in lateral wall displayed in 4-chamber (upper) and short-axis (lower) views.

**Figure 5:**
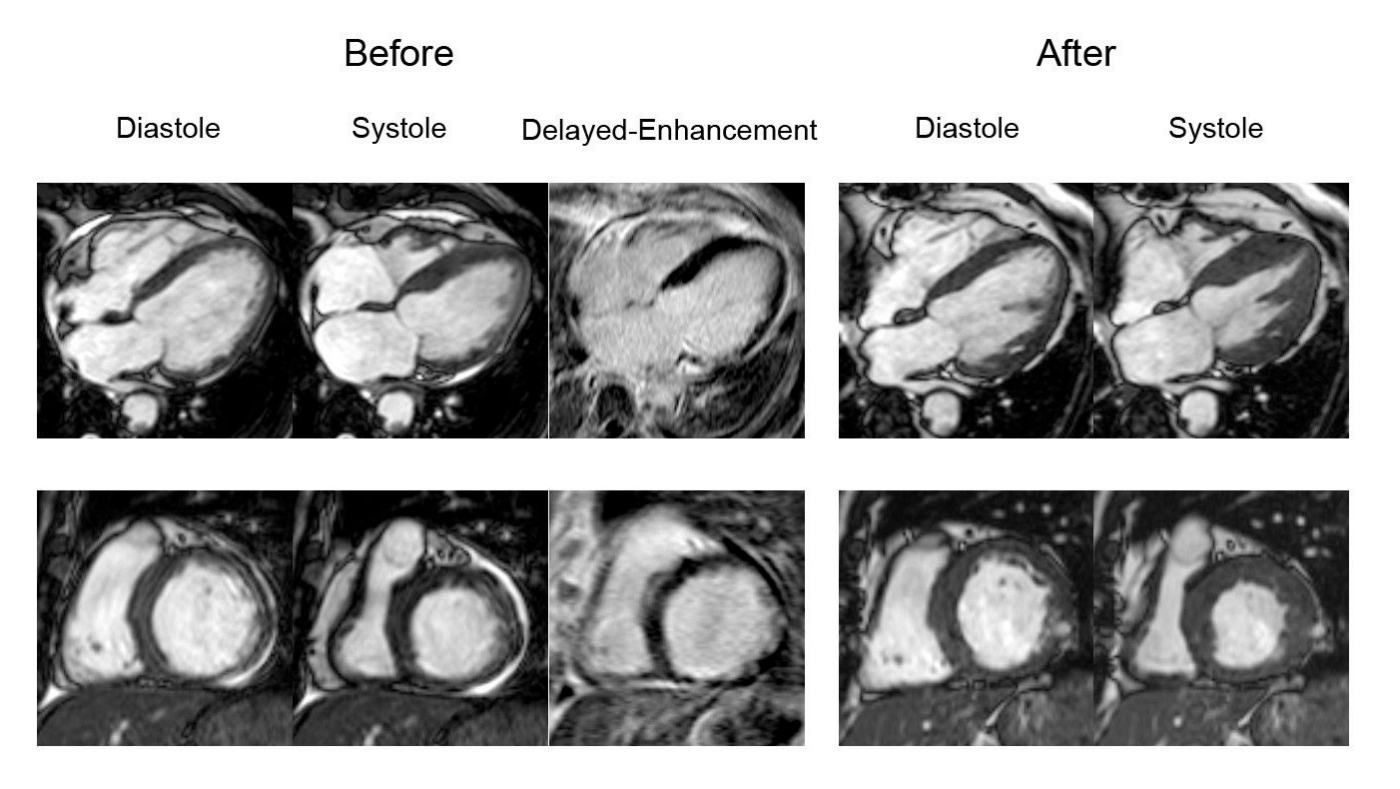
End-diastolic, end-systolic and LGE images before CABG and end-diastolic and end-systolic images after CABG of a patient with wall thinning and LGE area 1–25% in inferolateral wall displayed in 4-chamber (upper) and short-axis (lower) views.

We also tested ROC curve for ratio of LGE area and non-LGE area which was found to have area under curve, sensitivity, specificity, positive and negative predictive values of 0.805 (using cut off level 32.02%), 74.6%, 79.4%, 70.0% and 82.9% respectively. In the subgroup with LGE area 25–75%, area under curve of LGE area and total myocardial area versus LGE area and non-LGE area were exactly similar (0.638 for both methods). Since the overall area under curve of LGE area and total myocardial area had a better area under curve than LGE and non-LGE area, we used ratio of LGE and total area for further analysis. Comparison of ROC curves (Figure 2) showed that LGE area can predict wall motion improvement better than EDWT and resting wall motion grade (p < 0.001). The diagram shown in Figure 3 summarizes the additive value of LGE in the prediction of improved wall motion after CABG. If we used only EDWT, 144 segments with EDWT < 5.5 mm (26.1%) showed improved wall motion whereas 339 segments with EDWT ≥ 5.5 mm (50.1%) did not show evidence of improved wall motion. This results in a predictive accuracy of 60.6%. Adding information from LGE images will decrease the number of segments with a false prediction from 483 to 127 segments. Figures 4 and 5 show examples of 2 patients who had left ventricular wall thinning. The patient who had a large LGE area showed no evidence of improvement (Figure 4) whereas wall motion improvement was demonstrated in another patient who had a small LGE area (Figure 5).

### Prediction of LVEF improvement

Among 44 patients who had follow-up data, 22 patients (50%) had LVEF improvement defined as an increase in LVEF of at least 5%. Univariate analysis demonstrated that only baseline NYHA class, average LGE area and percent of segments with wall motion improvement are predictors of LVEF improvement (Table 5). There was no significant difference in number of diseased vessels and number of grafts between patients with and without LVEF improvement. There were also no significant differences in percent of segments with wall thinning (EDWT < 5.5 mm) and percent of segments with LGE area between patients with and without LVEF improvement. However, there were some trends of a higher chance of LVEF improvement in patient with fewer segments with wall thinning or LGE area.

**Table 5:** Univariate analysis of predictors for left ventricular ejection fraction improvement after CABG

Parameters	Left Ventricular Ejection Fraction Improvement		P value
	
	Yes (N = 22)	No (N = 22)	
Male (%)	21 (95.5)	20 (90.9)	0.550
Age (years)	59.4 ± 8.8	61.1 ± 9.9	0.566
NYHA class	2.82 ± 0.4	2.23 ± 0.53	< 0.001
History of MI (%)	14 (63.6)	16 (72.7)	0.517
Q wave from ECG (%)	8 (36.4)	11 (50)	0.361
Number of diseased vessels	3.7 ± 0.8	3.2 ± 0.9	0.075
Number of grafts	3.7 ± 0.8	3.3 ± 0.8	0.130
LVDD (mm)	59.7 ± 7.2	62.9 ± 9.1	0.203
LVSD (mm)	49.2 ± 8.8	52.8 ± 11.2	0.245
LVEDV (ml)	174.1 ± 53.4	191.5 ± 65.1	0.338
LVESV (ml)	109 ± 54.7	129.9 ± 64.6	0.254
Baseline LVEF (%)	40.2 ± 12.9	35.5 ± 13.2	0.244
Average LGE area (%)	32.9 ± 20	43.3 ± 18.6	0.008
Average EDWT (mm)	5.59 ± 1.5	5.58 ± 1	0.978
Percent of segments with wall thinning*,**	37.1 ± 19.8	46.8 ± 19.1	0.110
Percent of segments with LGE area*	51.4 ± 24.6	59.8 ± 24.1	0.264
Percent of segments with wall motion improvement (%)*	62.1 ± 22.4	20.5 ± 10.4	< 0.001

LVDD = left ventricular diastolic diameter, LVDS = left ventricular systolic diameter, LVEDV = left ventricular end-diastolic volume, LVESV = left ventricular end-systolic volume, LVSV = left ventricular stroke volume, LVEF = left ventricular ejection fraction, MI = myocardial infarction, NYHA = New York Heart Association, LGE late gadolinium enhancement

* Percent of segments = number of segment with feature divided by total analyzable segments

**wall thinning = EDWT < 5.5 mm

**Figure 6:**
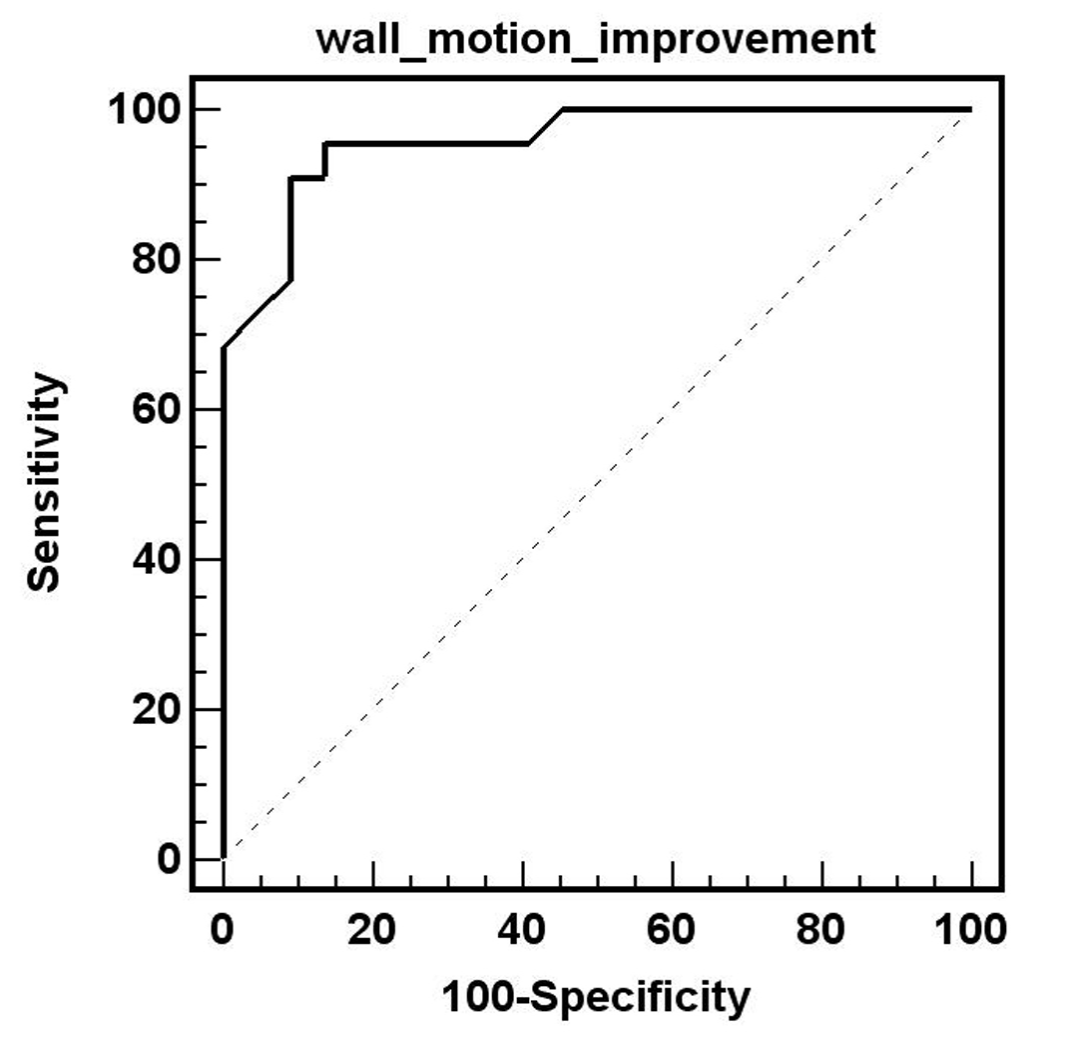
ROC curve of the prediction of an improvement of left ventricular ejection fraction of at least 5% by number of segments with wall motion improvement.

Logistic regression analysis showed that only the number of segments with wall motion improvement (Odds ratio 1.276, 95% CI 1.04–1.567, p = 0.020) remained in the final equation. ROC analysis demonstrated the area under the curve of 0.958 with the cut off at 26% (patients needed to have an improvement of wall motion abnormality of more than 26% of dysfunctional segments in order to have LVEF improvement of at least 5%) which results in a sensitivity of 95.5% and specificity of 86.4% (Figure 6). For percent of segments with wall thinning, using the cut off of ≤25%, the sensitivity, specificity and area under curve were 45.5%, 86.4% and 0.640 respectively. For the cut off of ≤37.5% of segments with LGE area, the sensitivity, specificity and area under curve were 36.4%, 86.7% and 0.591 respectively.

## Discussion

The main result of this study showed that extent of LGE area is the most important predictor for recovery of regional contractility after revascularization. EDWT and resting wall motion grade can also predict recovery of regional dysfunction but to a much lesser extent.

Increased signal intensity on LGE images is basically related to the altered sarcolemmal membrane integrity during acute injury and extracellular matrix structure as well as an expanded volume of distribution of gadolinium in scar tissue late after injury [11]. LGE is a very accurate technique in the assessment of acute and chronic myocardial infarction [12], determining infarct size after angioplasty [13] and is superior to SPECT in detecting myocardial necrosis after reperfusion in acute myocardial infarction [14]. LGE can be used to differentiate different etiologies of cardiomyopathy [15]. Although infarct mass calculated from LGE may be slightly decreased early after myocardial infarction [16], this technique has been shown to be highly reproducible [17]. Among 181 segments with subendocardial infarction identified by CMR, SPECT could detect an abnormality in only 47% [18]. Unrecognized myocardial infarction has been detected more than expected in a high-risk group with this technique [19]. It provides prognostic data both in patients with ischemic [20] and nonischemic cardiomyopathy [21]. Because of its high spatial resolution, reproducibility of functional CMR imaging is much better than those derived from echocardiography [22]. Therefore, CMR should be the appropriate technique for the assessment of the extent of LGE area, EDWT and global and regional left ventricular function.

It has been shown that LGE can predict improvement of regional wall motion after occlusion of the left anterior descending artery in dogs [23]. Earlier studies have shown that the best predictor of improvement of left ventricular function after revascularization is the extent of myocardial infarction less than 25% of left ventricular wall thickness [11,24]. The positive and negative predictive value of LGE area less than 25% of wall thickness in the prediction of recovery of regional function were 71 and 79% and none of 57 segments with LGE area more than 75% had an increase contractility after revascularization [6]. In our study, sensitivity and specificity of 25% of LGE area in identifying improvement of regional contractility were 73.2% and 83.2% respectively.

It has been shown that EDWT < 6 mm from echocardiogram virtually excludes the potential for recovery of function and EDWT > 6 mm had a sensitivity and specificity of 94% and 48% respectively for recovery of function [3]. Reversible wall thinning in hibernation can be predicted by CMR even in the patient with ejection fraction less than 30% [25]. In an CMR study, only 9.6% of segments with EDWT < 5.5 mm showed improvement in contractility after revascularization. In contrast, 48% of segments with preserved EDWT did not improve, thus indicating that EDWT should not be a good predictor for functional recovery [4]. Our study demonstrated that with the cut off of 5.5 mm, EDWT had a sensitivity and specificity of 70.1% and 81.5% in the prediction of functional recovery. Functional recovery was demonstrated in 26% of segments with EDWT < 5.5 mm and 49.9% of segments with EDWT ≥ 5.5 mm. LGE area is a better predictor for functional recovery than EDWT and has a good predictive power even in patients with an EDWT < 5.5 mm. The area of viable myocardium can be thinned and retain reserve for functional recovery after revascularization [26]. Theoretically, the area of thinned and viable myocardium can be developed during the process of ventricular remodeling after an acute event. It has been shown that assessment of ratio of non-viable to viable myocardium may have additional power for the prediction of functional recovery [26,27]. From our additional analysis, we found that the 2 methods were not different.

Dobutamine CMR can also be used to predict recovery of wall motion after revascularization. Recent data suggested that it may be even better than LGE [28] since low dose dobutamine can assess contractile reserve by demonstrating an increase in systolic wall thickness at low dose. LGE do not provide data on contractile reserve. However, earlier studies [29] showed that dobutamine CMR had a lower sensitivity for the prediction of recovery of wall motion especially in patients with severe left ventricular dysfunction.

We demonstrated that increased contractility of at least one-fourth of dysfunctional segments is needed in order to improve overall left ventricular function. The use of LVEF improvement of at least 5% was based on previous publications [30,31]. This is independent of other factors such as baseline left ventricular function, NYHA functional class, and left ventricular volume. Our findings are different from a previous report which indicated that end-systolic volume is the most important factor for the prediction of overall improvement of left ventricular function after revascularization compared to results of dobutamine stress echocardiography [30]. The differences may be related to the technique for the assessment of left ventricular function. CMR should provide more accurate data than echocardiogram. Another difference may be related to a less severe left ventricular dysfunction in our study. Patients with coronary artery disease and left ventricular systolic dysfunction had a high mortality rate during and after CABG as demonstrated in this study with 3 patients dying during hospital admission and 3 dying within 6 months of follow-up. Therefore, an appropriate strategy for the selection of patients for CABG is essential.

### Study limitations

There are some limitations to this study. Firstly, Results of this study are based on follow-up data at 4 months after CABG. However, recovery of left ventricular dysfunction may be delayed up to 14 months after revascularization [32]. We did not assess LGE during follow-up visits. Therefore, we cannot estimate the number of patients who might have perioperative myocardial infarction and no improvement of regional wall motion. Secondly, we did not analyze the relationship between the bypassed vessel and segments with improved wall motion. We thought that in patients with chronic CAD, there are complex collateral circulations and therefore the bypassed vessel may provide blood supply to the myocardial area in other coronary territories as well. Thirdly, this study limits revascularization by CABG and not percutaneous coronary intervention. Fourthly, the sample size is relatively small for the assessment of overall improvement of patients or predictors for poor outcome of CABG. Notwithstanding, our primary objective was the prediction of segmental improvement. Lastly, we do not have data on perfusion imaging or follow-up for bypass graft patency.

In conclusion, LGE and EDWT are independent predictors for functional recovery after revascularization. However, LGE is a more important factor and independent of EDWT.

## Competing interests

The authors declare that they have no competing interests.

## Authors' contributions

RK participated in conception, design, obtained funding, and drafted manuscript. PL involved in provision of patients. AM, PS and VC participated in analysis and interpretation of data, and involved in drafting manuscript. SU participated in statistical analysis. All authors read and approved the final manuscript.
